# Completion pneumonectomy: a valuable option for lung cancer recurrence or new primaries

**DOI:** 10.1186/s12957-018-1398-2

**Published:** 2018-05-28

**Authors:** Dragan Subotic, Laureano Molins, Ivan Soldatovic, Dejan Moskovljevic, Lucia Collado, Jorge Hernández

**Affiliations:** 1 Clinic for Thoracic Surgery, Clinical Center of Serbia, 26, Koste Todorovica, Belgrade, 11000 Serbia; 2 School of Medicine, University of Belgrade, Belgrade, Serbia; 3 Department of Thoracic Surgery, Hospital Clinic, Barcelona University, Barcelona, Spain; 4 Institute for Medical Statistics, School of Medicine, University of Belgrade, Belgrade, Serbia; 5 Department of Thoracic Surgery, Hospital Sagrat Cor, Barcelona University, Barcelona, Spain

## Abstract

**Background:**

The preoperative selection of patients with lung cancer recurrence remains a major clinical challenge. Several aspects of this kind of surgery are still insufficiently evidence-based, with only a few series with more than 50 patients.

**Methods:**

A retrospective study on 29 patients who underwent a completion pneumonectomy for postoperative lung cancer recurrence or new primary was done in the period between October 2004 and December 2015. Inclusion criteria include complete (R0) first and second resections, histologically proven recurrent or new malignancy, complete pathohistological report after both operations, and exact data about the treatment outcome at the time of the last contact with patients or their families.

**Results:**

There were 25 (86.2%) males and 4 (13.8%) females (M:F 6.2:1). In 13/29 patients, the interval between the first and second operations was less than 2 years, while in the remaining 16 patients, it was longer than 2 years. Concerning the operative stage distribution, stage I was more frequent after the first operation (44.8 vs. 22%), while stage III was dominant after the second operation (40.7 vs. 10.3%). The same tumor histology after the first and second operations existed in 24 (82.8%) patients. Adjuvant treatment was given to 53.6% of patients after the first and to 45.5% of patients after the second operation. The overall 5-year survival was 30%, median survival being 35 ± 16.9 months (1.896, 68.104 95% CI). A median survival of patients in post-surgery stage I after re-do surgery was better in comparison with that in higher stages (35 ± 22.6 vs.17.2 ± 15.1 vs. 21 ± 6.7 months, *p* > 0.05). Patients with the same tumor type at both operations lived significantly longer (median survival 48 ± 21.5 vs. 7.7 ± 1.9 months) than patients with different tumor histology after the second operation. Patients under 60 years (42.9%) lived longer than patients older than 60 years (median survival 69 ± 4.5 vs. 17.2 ± 14.3 months). The Cox regression analysis revealed only the disease stage at first operation and the same/different tumor histology as significant prognostic factors. One patient died from cardiac insufficiency caused by bronchopleural fistula (3.4% operative mortality). Operative morbidity was 34.4%.

**Conclusion:**

Completion pneumonectomy may be a reasonable option for postoperative lung cancer recurrence or new primaries only in carefully selected patients, in whom the potential oncological benefits overweigh the surgical risk.

## Background

Postoperative tumor recurrence, occurring in 30–77% of patients, is the main cause of a poor long-term survival after lung cancer surgery, still remaining less than 50% [1]. Surgery, in the form of a completion pneumonectomy, is possible in up to 4% of patients with loco-regional recurrence or new primaries [2]. Loco-regional recurrence, occurring in 4.6–24% of patients after complete resection [3], remains a major challenge from the standpoint of optimal diagnostic and therapeutic approach. The most frequent surgical option in these patients is a completion pneumonectomy, meaning removal of the remaining lung after the first resection, usually after lobectomy or bilobectomy, less frequently after a sublobar resection. Other therapeutic options are definitive chemotherapy and radiation therapy, either alone or combined [4, 5].

Patient selection for this kind of surgery is still insufficiently evidence-based. There are only a few series with more than 50 patients, focused more to the appropriateness of this operation from the standpoint of a high operative risk than from the standpoint of the oncological benefit.

The aim of the study is to assess the treatment outcome after completion pneumonectomy for postoperative lung cancer recurrence or new primary by analyzing data from two high-volume surgical centers.

## Methods

The analysis represents the two-institutional experience with 29 patients who underwent a completion pneumonectomy for postoperative lung cancer recurrence or new primary in the period between October 2004 and December 2015. Inclusion criteria for completion pneumonectomy include potentially curative (R0) first resection for primary NSCLC, histologically proven recurrent or new malignancy and/or CT aspect of malignancy at the ipsilateral hilar or parenchymal level, no distant metastases, and no multilevel N2 disease. In most of the patients, the indication for completion pneumonectomy was established preoperatively. Only in patients with sublobar resection, this decision was determined during the operation, because of the anticipated lack of radicality or reexpansion problems. In patients with left-sided (upper or lower) lobectomy as the first operation, completion pneumonectomy was the only possible option, because an additional generous wedge resection would have been associated with major reexpansion problems. In the present series, there were no patients with a middle lobe recurrence with a favorable local situation in terms of oncological radicality, in whom it would be possible to avoid completion pneumonectomy.

The mediastinal re-staging was based on high-resolution CT and PET. Patients with PET-positive N2 disease underwent a bronchoscopic (EBUS or rigid bronchoscopy transcarinal or transtracheal needle-biopsy). Re-mediastinoscopy was not a part of mediastinal re-staging in this group of patients. Upfront surgery was offered only to biopsy-negative patients. Biopsy-positive patients underwent a chemotherapy (2-3 cycles) and in case of response to treatment or in the absence of the disease progression, re-do surgery was offered. Current study inclusion criteria include complete (R0) second resection, histopathologically proven recurrent or new malignancy, detailed postsurgical histopathological report, and exact data about the treatment outcome at the time of the last contact with patients or their families.

Exclusion criteria include (1) patients with otherwise resectable tumors, but whose general condition was too poor for major surgery and subsequent adjuvant therapy; (2) unclear local disease spread in terms of complete resection; surgery was indicated only if the risk of explorative thoracotomy was practically zero, because of a high probability of lung expansion problems and related complications; on the other hand, involvement of the pericardium and intrapericardial part of the pulmonary vein and/or artery (without extension to the myocardium), as well as involvement of the proximal part of the main bronchus (if a sleeve pneumonectomy is still possible) and diaphragm, were not arguments for rejection from surgery; only direct invasion of the myocardium, trachea, esophagus, aorta, circumferential invasion of the superior vena cava and spinal involvement were absolute contraindications for surgery; (3) predicted-postoperative FEV_1_ < 30% in patients with COPD or restrictive ventilatory disorders; and (4) oxygen consumption during effort < 10 ml/kg in patients with increased overall cardiorespiratory risk (previous coronary artery stenting or bypass surgery, serious cardiac rhythm disorders, with or without associated COPD).

The preoperative lung function and risk assessment before the second operation followed the ESTS/ERS criteria for fitness for surgery [6]. All patients underwent the calculation of the predicted-postoperative FEV_1_ before the second operation by using a perfusion lung scintigraphy. Having in mind a re-do surgery, only patients with ppoFEV_1_ ≥ 35% were included. Diffusion was measured in all patients irrespectively of the lung function after the first surgery. Only patients with DLco ≥ 50% at rest were included. Patients with moderate COPD and patients with any type of cardiac co-morbidity, irrespectively of the lung function results, underwent exercise testing with determination of the oxygen consumption (VO_2_). The VO_2_ value ≥15 ml kg^− 1^ min^− 1^ was set up as cut-off value for re-do surgery. All patients were included in the program of preoperative therapy according to institutional protocol. Pulmonary physiotherapy (bronchodilator aerosols in 0.9% NaCl solution, during 10 min through jet nebulisers) was performed in three daily sessions, 5 days a week with the duration according to need. In COPD patients, in addition to physiotherapy, intravenous bronchodilator therapy (theophylline derivatives 12.5 or 25 mg twice a day) combined with Berodual, Spiriva, or Symbicort (in case of the need for faster lung function recovery) was administered as well. Hyposaturation in the arterial blood at rest was a contraindication for surgery.

The re-staging of the mediastinum was based on high-resolution CT and PET. Mediastinoscopy was not done for mediastinal re-staging in the analyzed group. In all patients, preoperative bronchoscopy was done at least 7 days before the scheduled surgery, in case additional biopsies and histopathological analyses from the site of the anticipated bronchial cut were necessary. The final selection was more restrictive than for the first operation, mostly in relation to the patients’ age and general condition.

Modified Martini-Melamed criteria [7] were used to differentiate recurrent from new primaries: if the tumor of the same histology was located at the bronchial stump level or within the parenchyma, close to the margins of previous resections, it was classified as a local recurrence, even after more than 2 years after the first operation. Disease stage was determined by using the actual, seventh edition of TNM classification for lung cancer.

The policy for lymphadenectomy during the lung resection in all lung cancer patients comprised systematic lymphadenectomy from the supreme mediastinal level down to the pulmonary ligament level. All the fatty tissues were removed together with the lymph nodes from the paratracheal, pretracheal, retrocaval, aortico-pulmonary, subcarinal (including N3 nodes), and paraesophageal region. The minimal quality requirement was at least four different mediastinal nodal groups removed, in addition to those removed en block with the specimen.

In 19 patients, the bronchial suture was performed manually, cartilage-to-cartilage, membranous-to-membranous, by using a continuous PDS 2-0 with 1-3 single, reinforcing PDS 3-0 stitches. In 10 patients, the main bronchus was stapled by a bronchial TA stapler. In all patients with right-sided pneumonectomy after induction chemotherapy, the bronchial stump was routinely protected by the pericardial fat pad or, when poor-quality, by the diaphragm muscle. In patients undergoing a right pneumonectomy after the radiation treatment, pericardial fat pad was the first choice, followed by omentum flap (two patients).

The quantitative variables are presented as means (standard deviation) or medians (ranges), depending on data distribution. Statistical significance was calculated using the log rank test. All *p* values less than 0.05 was considered significant. Survival (including operative mortality) was calculated by the Kaplan–Meier method. Survival analysis included disease stage after the first and second operations, depending on T and N factors, tumor histology, age, and interval between the two operations. Cox regression model was used to assess the influence of particular risk factors to study endpoints.

There were 25 (86%) males and 4 (14%) females (M/F 6.2:1). The age of the patients (mean, range) was 60.2 ± 7.6 (49–76) years. In 13/29 patients, the interval between the first and second operations was less than 2 years, and in the remaining 16 patients, it was longer than 2 years. In five patients, the tumor histology after the second operation was different from the tumor type at the first operation, and in all of them, the interval between the two operations was longer than 30 months, thus classifying them as clearly, new primaries. So, 24 patients had loco-regional relapse.

## Results

### Group description

Right- and left-sided operations were equally distributed (15/14), in a similar way as upper and lower lobectomies as the first operations, in 10 (34%) and 11 (38%) patients, respectively and tumor histology, with squamous cell and adenocarcinoma existing in 14 (48%) patients each. Sublobar resections were initially done in 17% of patients, while a middle lobectomy was the first operation in 3 (10%) patients. The structure of the analyzed group is presented on Table 1.

**Table 1:** Structure of the analyzed group

	Postoperative T								Postoperative N						Postoperative disease stage						Adj.Th
	T1		T2		T3		T4		N0		N1		N2		I		II		IIIA		
	*n*	%	*n*	%	*n*	%	*n*	%	*n*	%	*n*	%	*n*	%	*n*	%	*n*	%	*n*	%	%
IOP	10	34.4	16	55.1	2	6.9	1	3.4	15	51.7	13	44.8	1	3.4	13	44.8	13	44.8	3	10.3	53.6
IIOP	7	24.1	8	27.5	8	27.5	4	20.9	11	37.9	10	34.5	6	20.6	6	20.6	10	34.4	11	37.9	45.5

*IOP* first operation, *IIOP* second operation, *Adj. Th* adjuvant therapy

In patients with loco-regional recurrence, tumor relapse at the hilar level existed in 19/24 (79.1%) patients. Of these, endobronchial tumor growth or stenosis by extrabronchial compression, as confirmed by bronchoscopy, existed in 13 patients. In the remaining patients, the recurrence occurred at the parenchymal level.

Concerning the operative stage distribution, the stage I was more frequent after the first operation (45 vs. 22%), whilst the stage III was dominant after the second operation (40.7 vs. 10.3%). The same tumor histology after the first and second operations in existed in 24 (83%) patients, while it was different in five (17%) patients. Adjuvant treatment was given to 53.6% of patients after the first and to 45.5% of patients after the second operation.

In no patients, the lung function after the first operation was within normal ranges. Purely restrictive ventilatory disorders existed in 8 (27.7%) of patients and mixed disorders in 7 (24.1%) patients, while in the remaining 14(48.2%) patients, light and light to moderate COPD was equally distributed. Combined COPD and cardiac comorbidity existed in 11 (37.9%) patients (cardiac rhythm disorders and myocardiopathy in 7 and 4 patients, respectively). Diabetes mellitus existed in 6 (20.6%) patients.

### Survival

**Fig. 1:**
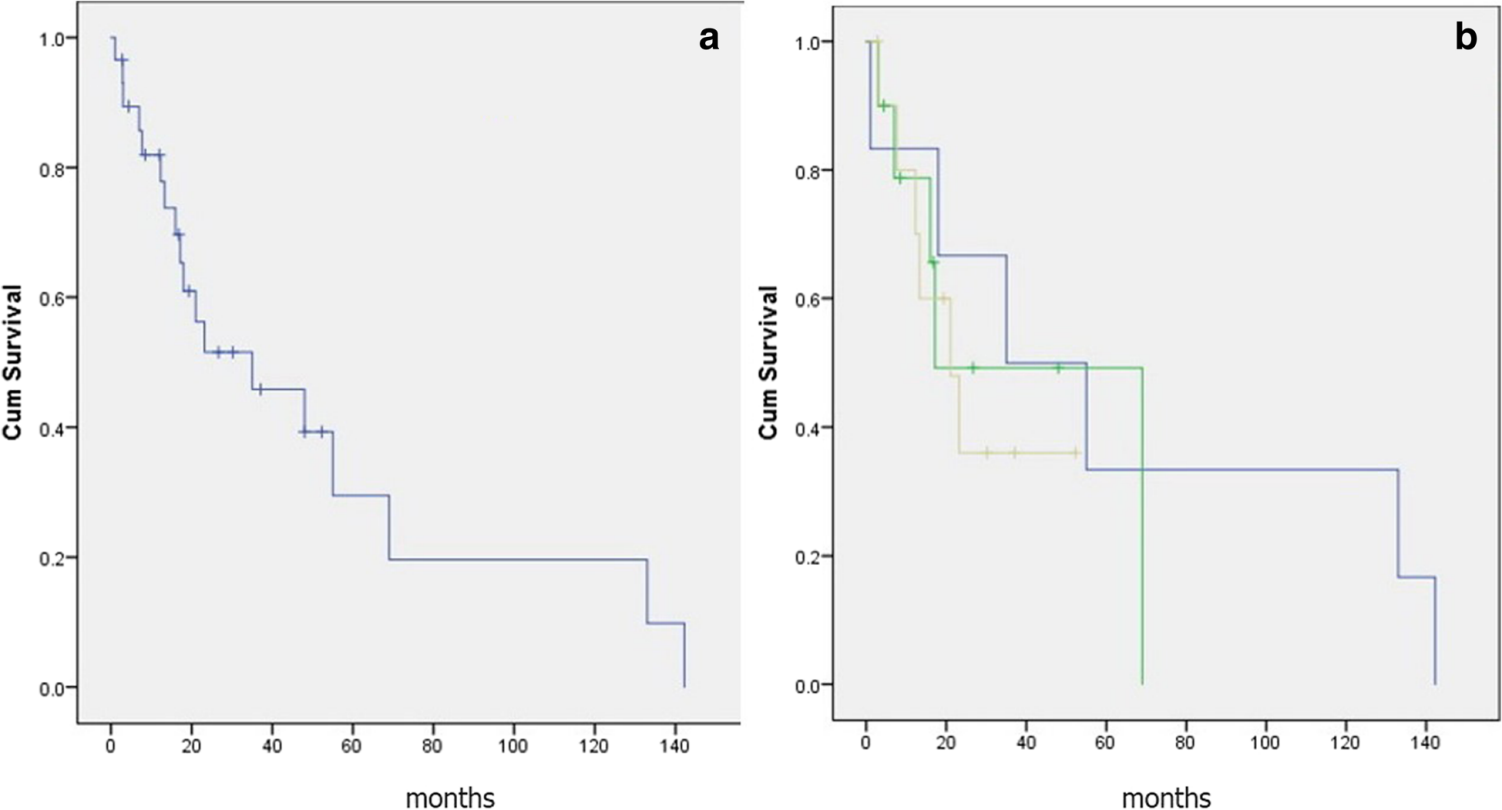
Survival after completion pneumonectomy. **a** Overall. **b** Depending of disease stage of the second operation. Blue, stage I; green, stage II; gray, stage III

The overall survival is presented in Fig. 1a. The 3- and 5-year survival was 53 and 30%, respectively. Median survival was 35 ± 16.9 months (1.9, 68.1 95% CI). A separate analysis including only patients with a loco-regional relapse demonstrated that a median survival was 48 months (5.8–90.1 CI 95%), while 3- and 5-year survival were 59 and 33%, respectively. The disease-free interval for the entire group was 30 (12.4–47.6) months.

The disease-free interval in patients operated in stage I was longer in comparison with patients operated in stages II and III—42(30.5–53.4) vs. 20 (14.1–25.8) months, respectively (*χ*^2^ = 0,813; *p* = 0,367).

Survival of patients depending on disease stage after the completion pneumonectomy is presented in Fig. 1b and Table 2. Despite a better median survival of patients in stage I (compared with stages II and III—35 ± 22.6 vs. 17.2 ± 15.1 vs. 21 ± 6.71 months), this survival difference did not reach the level of statistical significance. There was no significant survival difference depending on the length of interval between the first and second operations (median survival 55 ± 12.4 vs. 17.2 ± 1.5 months), with the cut-off interval set up at 31 months, based on median interval (Table 2).

**Table 2:** Survival (in months) depending on disease stage and interval between the operations

Op stage	Median	SE	95% CI		Mean	SE	95% CI	
I	35.0	22.65	0.00	79.4	64.03	24.41	16.18	111.88
II	17.20	15.17	0.00	46.93	39.97	11.84	16.75	63.19
III	21.0	6.76	7.74	34.25	27.75	6.30	15.38	40.11
	Survival according to interval between the two operations							
< 30 months	55.0	12.42	30.65	79.34	57.85	15.85	26.79	88.92
> 30 months	17.20	1.57	14.11	20.28	43.68	18.09	8.22	79.14

< 30 months, interval between the two operations shorter than 30 months; > 30 months, the same interval longer than 30 months

**Fig. 2:**
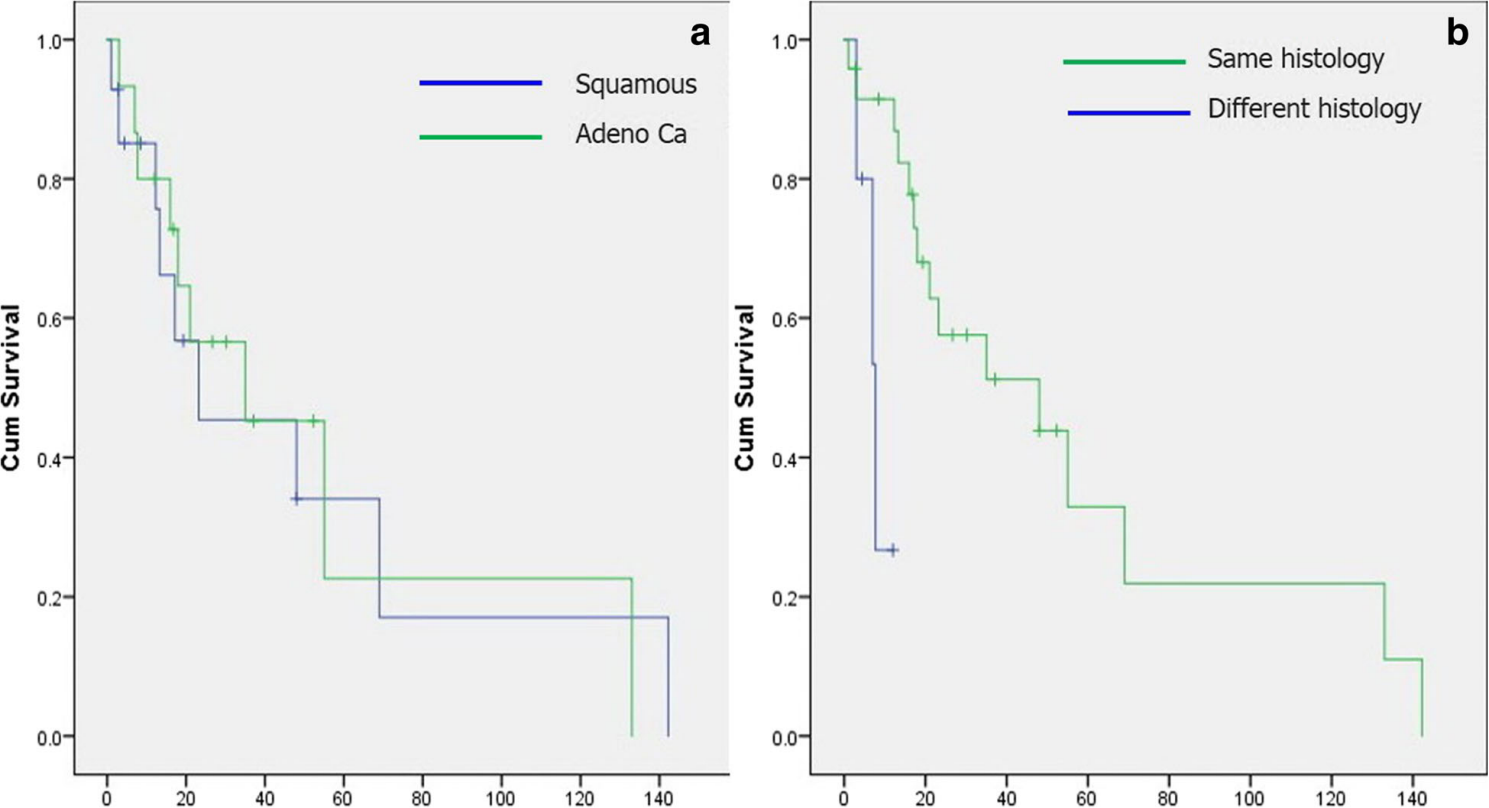
Survival depending of tumor histology. **a** Squamous cell carcinoma vs. adenocarcinoma; **b** same vs. different tumor type

There was no significant survival difference between patients with squamous and adenocarcinoma (Table 3, Fig. 2a). Patients with the same tumor type at both operations lived significantly longer (median survival 48 ± 21.5 vs. 7.7 ± 2 months) than patients with different tumor histology after the second operation (Table 3, Fig. 2b).

**Table 3:** Survival (in months) depending of tumor histology

Tumor type	Median	SE	95% CI		Mean	SE	95% CI	
Squamous	23.20	21.21	0.00	64.78	48.38	17.01	15.03	81.72
Adeno Ca	35.0	14.86	5.86	64.13	51.99	17.74	17.20	86.77
PH1 = PH2	48.0	21.5	5.85	90.14	55.61	12.90	30.32	80.90
PH1 ≠ PH2	7.7	1.98	3.80	11.59	7.72	1.46	4.84	10.59

PH1 = PH2, tumor histology was the same after both operations; PH1 ≠ PH2, tumor histology after the second operation was different vs. histology after the first operation

*PH1* pathohistology at first operation, *PH2* pathohistology at second operation

**Fig. 3:**
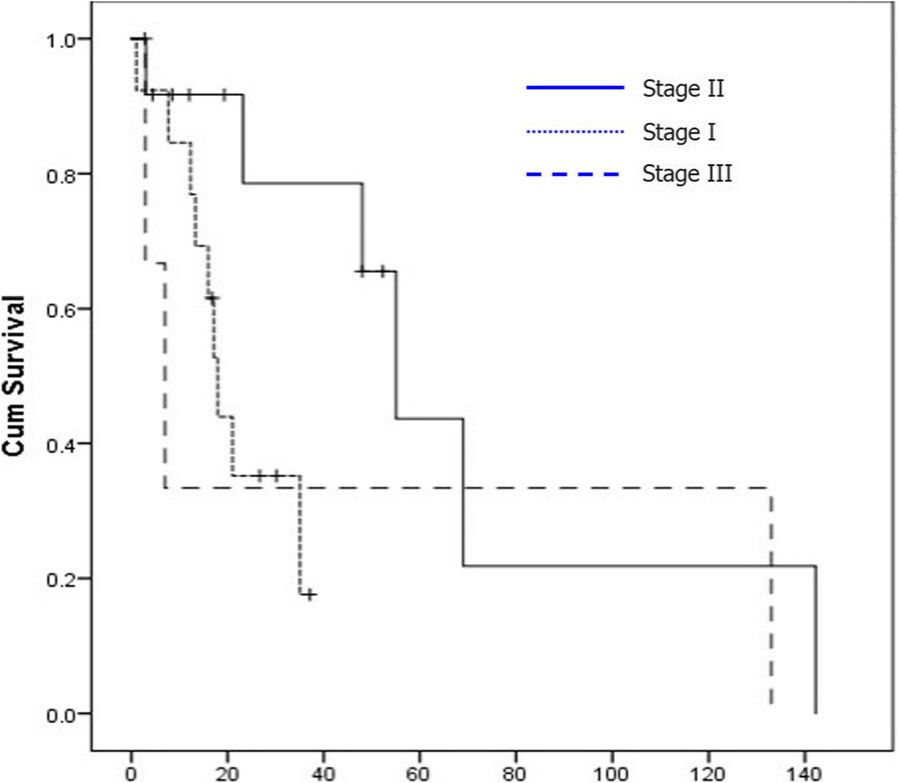
Survival depending of diseases stage of the first operation

**Fig. 4:**
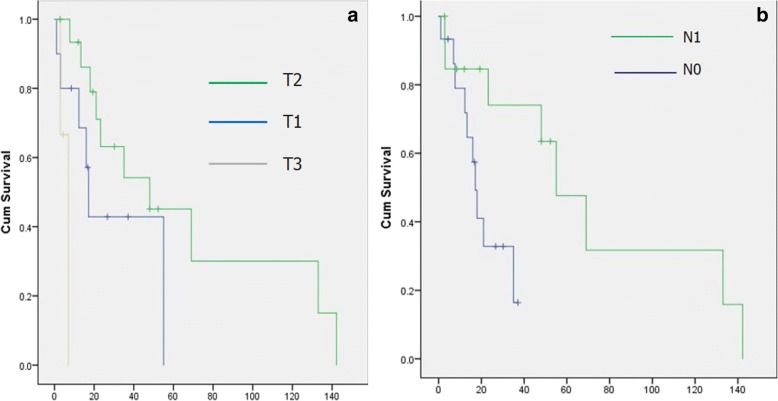
Survival depending of T (**a**) and N (**b**) factors at first operation

The median survival of patients with stages I, II, and III at first operation was 18 (95% CI 14.8–21.2) vs. 55 (95% CI 41.8–68.2) vs. 7 months (95% CI 0.43–13.5), respectively Table 4, Fig. 3). This survival difference was not statistically significant (*χ*^2^ = 4.38; *p* = 0.11). However, because of a small number of patients in stage III at first operation, a separate comparison between stages I and II revealed a significant survival difference (*χ*^2^ = 5.6, *p* = 0.01). Survival depending on T and N factors at first operation is presented on Fig. 4 and Table 4. Survival of patients with T2 tumors was significantly better than survival of patients with T1 tumors (*χ*^2^ = 11.1; *p* = 0.004) (Fig. 4a). Similarly, the median survival of patients with N0 lesions was inferior to survival of patients with N1 lesions 17.2 (13.9–20.4) vs. 55 months (41.5–68.4), respectively. This survival difference was statistically significant (*χ*^2^ = 4.3; *p* = 0.04) (Fig. 4b). Age of the patients was associated with significant survival difference in a way that patients under 60 years (42.9%) lived longer than patients older than 60 years (median survival 69 ± 4.5 vs. 17.2 ± 14.3).

**Table 4:** Survival (in months) according to stage, T and N factors of the first operation

Op stage	Median	SE	95% CI		Mean	SE	95% CI	
I	18.0	1.62	14.82	21.18	21.48	3.34	14.92	28.04
II	55.0	6.73	41.8	68.19	67.67	19.44	29.56	105.78
III	7.00	3.34	0.43	13.56	47.63	42.7	0.00	131.3
T1a + T1b	17.20	1.64	14.33	20.0	29.66	8.45	13.1	46.22
T2a + T2b	48.0	19.83	9.11	86.88	65.52	16.25	33.65	97.38
T3 + T4	7.0	0.00	–	–	5.63	1.57	2.54	8.72
N0	17.20	1.66	13.94	20.45	20.55	3.27	14.20	26.89
N1 + N2	55.0	11.74.	31.98	78.01	71.31	18.06	35.92	106.71

### Prognostic factors

The Cox regression analysis included age, disease and nodal stage at first operation, tumor histology (same vs. different), and interval between the two operations. Only disease stage at first operation (HR = 3.44, 95% CI, df = 1, sig. = 0.06) and same/different tumor histology (HR = 7.85, 95% CI, df = 1, sig. = 0.005) were identified as significant prognostic factors.

### Operative mortality and morbidity

One patient died from cardiac insufficiency caused by bronchopleural fistula, thus constituting 3.44% 30-day operative mortality. Postoperative complications were registered in 10 (34.5%) patients. Postoperative bleeding (successfully solved by rethoracotomy) and bronchopleural fistula occurred in two (7%) patients each. One of two patients with bronchopleural fistula was the only one who died within 30 days postoperatively. There was one patient with pleural empyema without bronchopleural fistula and another one with postoperative pneumonia of the remaining lung. In this patient, pneumonia resolved after 7 days of parenteral antibiotic therapy. The heart arrhythmia requiring medical treatment occurred in four (14%) patients. Bronchopleural fistula occurred in two patients: the first one was a patient aged > 65 years with generalized disease that was unrecognized at the time of surgery and this patient died within the first 30 postoperative days; the other one was a younger patient operated after previous chemotherapy, in whom extrapleural lung liberation and excessive peribronchial dissection were done. The fistula occurred despite the bronchial stump protection by the pericardial fat pad. In this patient, the subsequent empyema was put under control by initial chest tube aspiration followed by repeated thoracenteses after the chest tube removal.

The 90-day mortality did not differ from the 30-day mortality, as checked and confirmed during regular outpatient controls according to widely accepted schedule.

## Discussion

### Survival

The 30% overall 5-year survival of patients in the current study fits within the range of reported survival rates. According to data from 10 studies published before 2007 [2, 8–16], each including more than 30 operated patients, 5-year survival was under 30% in four studies, between 30 and 40% in additional three studies, while it was over 40% in three studies (44, 44.5, and 57%). In a multicentric study on 165 patients, published in 2012, the 5-year survival was 48.9 and 23.9%, for squamous cell and adenocarcinoma, respectively [17]. The reason for better long-term survival of patients in the present study, compared with studies with survival rates under 25%, is the higher proportion of stage III patients in these studies (66 vs. 40.7% in the present study). Stage-related survival in the analyzed group confirmed the significance of the disease stage, whose influence remains inconsistent throughout the literature. In the present study, despite a better survival in the stage I, the survival difference was not statistically significant, like in the aforementioned study of Guggino et al. (67.5 vs. 33.3% for stages I and II, respectively) [2]. Unlike that, in the study of Chataigner et al. [18], survival of patients with stage I and II disease was significantly better than in patients with stage III disease (56 vs. 17%; *p* = 0.02).

Our finding of significant survival difference between recurrent and second primary tumors should be taken with caution, because of the small number (17.25%) of patients with clear criteria for second primaries. Such a finding is not in line with literature data, varying between no survival difference (38 vs. 41%) [18], through slightly better (44.6 vs. 29.2%) [2] to significantly better survival for second primaries, with only 10% of the patients with a recurrent carcinoma surviving 21 months [16]. Some other results confirm the same survival trend [9]. Such an inconsistence in the reported data is probably due to different criteria for cancer recurrences and new primaries.

Although some data suggest a poor prognosis of recurrent adenocarcinoma vs. squamous cell carcinoma, like in the aforementioned multicentric study [17], there is not enough evidence for this factor to be taken into account as essential for preoperative patient selection. In the present study, survival difference depending on tumor type was not significant. In the present study, despite a certain trend in favor of interval between the two operations shorter than 31 months (mean survival 57.8 ± 15.8 vs. 43.6 ± 18.1 months), this survival difference was not significant. In one of the few studies dealing with this factor, longer interval was associated with better survival, but again without statistical significance (35.4 vs. 54.6% 5-year survival for intervals < 2 and > 2 years, respectively) [2]. Unclear prognostic significance of this factor additionally complicates the preoperative selection.

As the influence of the disease stage and other relevant factors at the time of initial surgery are not sufficiently addressed in the literature, this aspect was separately analyzed in the present study. Our data suggest that, if patients with a stage I get the lung cancer recurrence, they will do worse than those with recurrence after the stage II disease at first surgery. This finding is less surprising having in mind that the 10–15% reported incidence of local recurrence in early-stage lung cancer [19]. In addition, a high recurrence rate was reported in patients with stage I after complete resection (25–50%) [20]. However, based on the analyzed data, an appropriate explanation for the obtained stage-related survival could not be given. The size of the analyzed series is quite limited, although it is also debatable whether the greater series would give some additional answers or clarifications. Including some of biochemical tumor markers into the analysis could possibly be of benefit for future studies of this problem.

### Adjuvant therapy

In the present study, adjuvant treatment was given to 53.6% of patients after the first operation and to 45.5% of patients after the second operation. In fact, one important point in the preoperative selection was the likelihood that the patient will be fit for subsequent adjuvant treatment. Based on the promising results of five largest adjuvant chemotherapy trials with a confirmed survival benefit at 5 years [21–23], in terms of recurrence prevention, it still remains to be confirmed whether such a treatment would be also appropriate in patients with visceral pleural, intravascular, and lymphatic invasion, irrespectively of nodal stage, both after the initial and re-do surgery.

### Operative morbidity and mortality

The 3.4% mortality and 34.5% complication rates in the present study are strongly in favor of the appropriateness of this type of surgery, provided the patient selection is adequate. The main concern related to this operation has traditionally been the alleged high complication rate. However, based on ~ 12% reported operative mortality and < 40% operative morbidity in the last 15 years, in the majority of series, it is clear that these complication rates are comparable with those after standard pneumonectomy [24]. The 6.9% bronchopleural fistulas in the present study falls within the range of the reported rates, being 7–13.3% in the majority of the series. Despite a sufficient number of surgical series, the reported data are of limited value in terms of operative risk prediction. Even in the biggest series, either no significant factors of complications were identified or factors were found as significant only by univariate analysis, like for example, predicted-postoperative (ppo) FEV_1_ < 50% [17]. Our experience, supporting many other reports [25–27], is strongly in favor of bronchial stump protection, especially for right-sided operations. In the present series, pericardial fat, diaphragm, and omental flaps were used whenever possible. We also want to point out the risk of intraoperative death, reaching 5.3%, with the main causes of death being injury of the great vessels of the heart, usually due to major pleural or pericardial adhesions as a consequence of the first surgery [28]. Our policy to avoid such incidents, as previously reported by others, was the early opening of the pericardium, rather than dissection around shortened vessels surrounded by thickened peribronchial and perivascular tissue.

### Study limitations

The major limitation of the current study is the small number of included patients, determined by strict selection criteria for this kind of surgery. The patient number was additionally restricted by the need to include well-matched groups from two institutions.

Another limitation is survival analysis after two operations in different settings. By using the date of either the initial or re-do surgery as “time zero,” some pitfalls will appear. As we recently discussed this problem, in the first case, there will be a period of no failures and no censored observations prior to the earliest time of the second surgery. In this situation, the proportional hazard evaluation is violated and *p* values will probably be inadequately smaller [29]. In the second case, in patients with longer period between the two surgeries, the survival could be biased downward compared with patients with earlier re-operation, even if survival from the first surgery was the same. This is a widely accepted approach, and patients with a longer interval between the completion of the initial treatment and progression usually have a better post-progression prognosis. The aforementioned pitfalls were counterweighted by performing both methods of survival analysis.

## Conclusion

The obtained results confirmed that completion pneumonectomy may be a reasonable option in case of postoperative lung cancer recurrence or new primaries, but only in carefully selected patients. In this group of patients, the oncological benefits should overweigh the operative risk. In addition to standard selection criteria, the ability of the patient to sustain a subsequent adjuvant therapy should be also taken into consideration.
